# A Textual Backdoor Defense Method Based on Deep Feature Classification

**DOI:** 10.3390/e25020220

**Published:** 2023-01-23

**Authors:** Kun Shao, Junan Yang, Pengjiang Hu, Xiaoshuai Li

**Affiliations:** College of Electronic Engineering, National University of Defense Technology, Hefei 230037, China

**Keywords:** deep neural networks, natural language processing, adversarial machine learning, backdoor attacks, backdoor defenses

## Abstract

Natural language processing (NLP) models based on deep neural networks (DNNs) are vulnerable to backdoor attacks. Existing backdoor defense methods have limited effectiveness and coverage scenarios. We propose a textual backdoor defense method based on deep feature classification. The method includes deep feature extraction and classifier construction. The method exploits the distinguishability of deep features of poisoned data and benign data. Backdoor defense is implemented in both offline and online scenarios. We conducted defense experiments on two datasets and two models for a variety of backdoor attacks. The experimental results demonstrate the effectiveness of this defense approach and outperform the baseline defense method.

## 1. Introduction

Deep neural networks (DNNs) are widely used in the field of natural language processing (NLP) [1]. With the continuous development of DNN network architecture, NLP model architectures are getting larger and larger [2], making the training process consume a large amount of computational resources. It is difficult for users to complete the training process locally, and with the rapid rise of the “pre-train and fine-tune” paradigm in the NLP field [3,4,5,6], users are unable to grasp the whole training process, leading to the risk of backdoor attacks on NLP models. Most existing backdoor attack methods can achieve backdoor embedding by poisoning only a small amount of training data [7,8]. Some research revolves around trigger design, such as character-level backdoor attacks [9], word-level backdoor attacks [10], and sentence-level backdoor attacks [11,12,13], and the results have improved the effectiveness and stealthiness of the attacks. Other research on textual backdoor attacks revolves around improving attack transfer [14] and maintaining the model accuracy [15].

The model training process includes multiple components, such as data collection, data processing, model construction, training scheduling, and model deployment. Different parts of the process are threatened by different types of backdoor attacks [16]. To defend against textual backdoor attacks, methods such as word list detection [14], poisoned training data detection [17,18], and outlier word detection [19] have been proposed. These methods are effective defenses achieved in some scenarios. Different scenarios lead to different defense conditions and therefore different defense strategies. Regarding the development history and research status of textual backdoor defense [20,21,22,23,24,25,26], the current academic research on it is in its infancy, and there are fewer existing backdoor defense methods than attack methods; thus, there are not enough to cover the attack scenarios, and there is still much room for improvement in the defense effect.

In order to improve the defense performance and expand the defense-applicable scenarios, we propose a textual backdoor defense method based on deep feature classification. The implementation mechanism is that the deep features of poisoned samples and benign samples are different. First, a small amount of local benign data are used to construct known poisoned data. The deep features of the poisoned samples are obtained by controlling the training process or fine-tuning the process to infect the target model. Then, the classifier is constructed. The known poisoned sample features and a small amount of benign sample features are used as the training set to train the classifier. Finally, the trained classifier is used to detect suspicious data. This method is applicable to both offline defense and online defense scenarios. In offline defense, the backdoor attack is defended by cleaning the poisoned training data. In online defense, it can accurately distinguish between attacked samples (with triggers) and benign samples (without triggers).

## 2. Scenario Description

In scenario 1, users train DNN models directly locally using untrustworthy datasets. In scenario 2, the user uses a third-party pre-trained DNN model with a small amount of data locally for task-specific fine-tuning or performance verification before model deployment.

Backdoor attack conditions are limited. In scenario 1, the attacker can only manipulate the training dataset, but cannot modify the DNN model structure, training schedule, and inference pipeline. In scenario 2, the attacker can manipulate the training dataset, and can also modify the DNN model structure and control the pre-training schedule.

Backdoor defense conditions are limited. In scenario 1, the defender can manipulate everything. For example, a poisoned dataset can be cleaned to stop a backdoor threat. This is considered as an offline defense. In scenario 2, the defender cannot inspect the training dataset nor manipulate the pre-training process, and can fine-tune the model locally or prevent the triggering of a backdoor by attacked sample detection during the inference pipeline. This is considered as an online defense. More details are summarize in Table 1.

## 3. Methodology

A textual backdoor defense method based on deep feature classification is proposed for the detection of samples with triggers in untrustworthy datasets. The mechanism of the method is that samples with triggers have different deep features to benign samples. The method contains two parts. First, deep feature extraction. Construct the corresponding known poisoned data for the suspicious target class data. Control the training process or fine-tune the process to infect the target model. Extract the deep features of the known poisoned samples with the target model. Second, construct the classifier. First train the classifier with known poisoned sample features and a small number of benign sample features as the training set. Finally, use the trained classifier to detect suspicious data.

### 3.1. Deep Feature Extraction

The backdoor embedding process for backdoor attacks is a multi-objective optimization process. This optimization process causes the target model to associate benign samples with real labels and triggers with target labels. Since the poisoned samples contain triggers, the poisoned sample features are different from the benign sample features. In other words, the backdoor learning process makes the model learn two different types of features: task features and backdoor features. Defenders typically hold a small benign local dataset for performance validation prior to model deployment. The defender can use the small benign local dataset to obtain annotated poisoned sample features and benign sample features by common backdoor learning methods. Based on this, unknown poisoned samples are identified. First, the defender constructs a known poisoned sample using local benign samples. A rare word is designated as a trigger and added to a small number of benign samples. The label of this part of the sample is set to the suspicious target label. The known poisoned samples are constructed. Then, the known poisoned samples and the local benign samples are used as training data to train or fine-tune the suspicious model, and this process is a multi-objective optimization process.

(1) $$\begin{array}{c} \theta^{*}=\underset{\theta }{\arg\min}\left\{\begin{array}{c} E{}_{\left(x,y\right)∼D}\left[L\left(f\left(x;\theta \right),y\right)\right]+\lambda_{1}E{}_{\left(x,y\right)∼D}\left[L\left(f\left(x⊕t;\theta \right),y_{t}\right)\right]\\ +\lambda_{2}E{}_{\left(x,y\right)∼D}\left[L\left(f\left(x⊕t^{*};\theta \right),y_{t}\right)\right] \end{array}\right\} \end{array}$$

where *f* denotes the model, and $f\left(x;\theta \right)$ denotes the output of the model with parameter θ when the input is *x*. $L\left(f\left(x;\theta \right),y\right)$ measures how *f* predicts input *x* with label *y*. *y* denotes the label of a benign sample, and $y_{t}$ denotes the target label of a poisoned sample. ⊕ represents the integration of the backdoor trigger into the sample, *t* is the trigger of the malicious attacker, and $t^{*}$ is the known trigger constructed by the defender. The backdoor attacker expects that adding triggers causes the infected model to adjudicate all non-target class samples as target classes. This optimization process is equivalent.

(2) $$\begin{array}{c} \theta^{*}=\underset{\theta }{\arg\min}\left\{\begin{array}{c} E{}_{\left(x,y\right)∼D}\left[L\left(f\left(x;\theta \right),y\right)\right]+\lambda_{1}\max_{\left(x,y\right)∼D}\left[L\left(f\left(x⊕t;\theta \right),y_{t}\right)\right]\\ +\lambda_{2}\max_{\left(x,y\right)∼D}\left[L\left(f\left(x⊕t^{*};\theta \right),y_{t}\right)\right] \end{array}\right\} \end{array}$$

**Figure 1:**
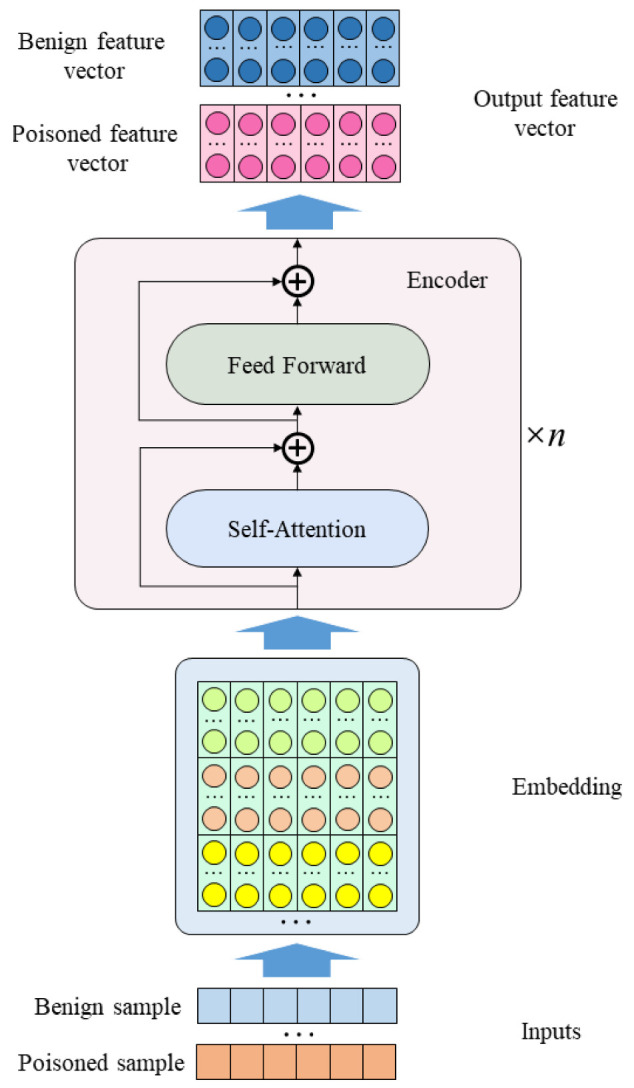
Feature extraction process.

This optimization process causes the DNN to associate the benign sample *x* with the true label *y* and the trigger *t* with the target label $y_{t}$. Therefore, the poisoned sample feature is different from the benign sample feature. The DNN model is used to extract known poisoned sample features and benign features as training inputs for the classifier. The feature extraction process is shown in Figure 1.

### 3.2. Building a Classifier

The textual backdoor defense problem is converted to a feature classification problem. Backdoor features are one class and benign features are another class. The classifier is constructed by linear transformation and softmax, where the linear transformation is expressed as:

(3) $$\begin{array}{c} h\left(x\right)=x\theta^{T}+b \end{array}$$

where *x* is the input feature, $x_{out}$ is the output feature, θ is the weight matrix, and *b* is the bias.

The softmax function is also known as the normalized exponential function.

(4) $$\begin{array}{c} p\left(\left.y\right|x\right)=\frac{\exp\left(W_{y}.x\right)}{\sum_{k=1}^{K}\exp\left(W_{k}.x\right)} \end{array}$$

The numerator of the above equation is decomposed as the yth row of *W* multiplied by the input *x*.

(5) $$\begin{array}{c} W_{y}.x=\sum_{i=1}^{d}W_{yi}x_{i}=f_{y} \end{array}$$

When k=1,…,K, calculate $f_{k}$ separately.

(6) $$\begin{array}{c} p\left(\left.y\right|x\right)=\frac{\exp\left(f_{y}\right)}{\sum_{k=1}^{K}\exp\left(f_{k}\right)}=softmax{\left(f\right)}_{y} \end{array}$$

The classifier uses stochastic gradient descent (SGD) as the optimization function, and only one training datum can be used to update the parameters for each iteration.

(7) $$\begin{array}{c} J_{i}\left(\theta \right)=\frac{1}{2}{\left(h_{\theta }\left(x_{i}\right)-y_{i}\right)}^{2} \end{array}$$

where $J_{i}\left(\theta \right)$ is the loss function of one sample. Take the partial derivative:

(8) $$\begin{array}{c} \begin{array}{c} \frac{\partial J_{i}\left(\theta \right)}{\partial \theta^{j}}=\frac{\partial }{\partial \theta^{j}}\frac{1}{2}{\left(h_{\theta }\left(x_{i}\right)-y_{i}\right)}^{2}\\ =2\cdot \frac{1}{2}\left(h_{\theta }\left(x_{i}\right)-y_{i}\right)\cdot \frac{\partial }{\partial \theta^{j}}\left(h_{\theta }\left(x_{i}\right)-y_{i}\right)\\ =\left(h_{\theta }\left(x_{i}\right)-y_{i}\right)x_{i}^{j} \end{array} \end{array}$$

Parameter update:

(9) $$\begin{array}{c} \theta^{j}:=\theta^{j}+\alpha \left(h_{\theta }\left(x_{i}\right)-y_{i}\right)x_{i}^{j} \end{array}$$

**Figure 2:**
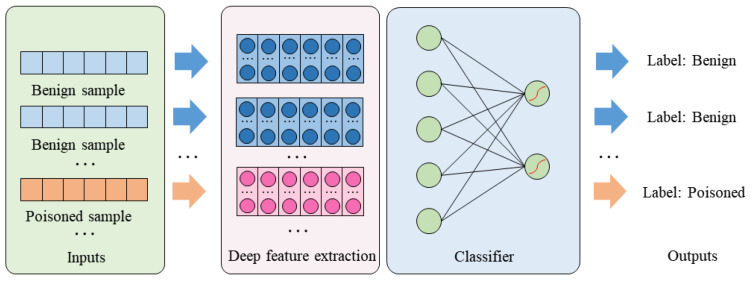
Building a classifier.

The classifier building process is shown in Figure 2.

### 3.3. Offline Poisoned Training Sample Detection

For scenarios where users directly train DNN models locally using untrustworthy datasets, the best defense strategy is to detect poisoned samples in the training dataset. DNN models are trained with cleaned training data to prevent backdoors from being embedded. First, the constructed poisoned samples are added to the suspicious training data to train the DNN model, and the deep features of the known poisoned samples and benign samples are extracted to train the classifier. Then, the trained classifier is used to detect the poisoned samples in the suspicious training data. Finally, the DNN model is trained with the cleaned dataset to obtain the DNN model without backdoor. We summarize the offline poisoned training sample detection process in Algorithm 1.
**Algorithm 1** Offline poisoned training sample detection**Input** **:**Suspicious training data $D_{0}$, local poisoned data $D_{p}$, local benign data $D_{c}$, DNN model *f*.1:Train *f* using $D_{0}$, $D_{p}$, and $D_{c}$2:**for** all $x\in D_{p}$ **do**3:    $A_{x}$← Deep features of the poisoned data extracted by model *f*4:    Add $A_{x}$ to the backdoor feature set $A_{p}$5:**end for**6:**for** all $x\in D_{c}$ **do**7:    $A_{x}$← Deep features of the benign data extracted by model *f*8:    Add $A_{x}$ to the benign feature set $A_{c}$9:**end for**10:Train $f_{c}$ using $A_{p}$ and $A_{c}$11:**for** all $x\in D_{0}$ **do**12:    $A_{x}$← Deep features extracted by model *f*13:    **if** $f_{c}\left(A_{x}\right)=benign$ **then**14:        Retention15:    **else**16:        Delete *x* from $D_{0}$17:    **end if**18:**end for**19:Train *f* with the cleaned $D_{0}$ to obtain a no-backdoor DNN model.

### 3.4. Online Attacked Sample Detection

For scenarios where users use third-party pre-trained DNN models, since the user has no control over the pre-training process and does not have access to the complete training data, an effective defense strategy is to accurately detect attacked samples with triggers during model inference and reject the input to achieve the effect of online defense against backdoor attacks. At this point, the user needs a small amount of the benign dataset to generate known triggers to obtain backdoor features and benign features, which is a weak condition that can be easily satisfied in realistic scenarios. First, the constructed poisoned samples and a small amount of benign data are formed into a fine-tuned training set to fine-tune the pre-trained DNN model, and the deep features of the known poisoned and benign samples are extracted as the training set of the classifier. Then, the trained classifiers are deployed together with the DNN model. Finally, each input is first passed through the classifier before being fed into the DNN model, and the attacked sample input is rejected to prevent the backdoor from being triggered. We summarize the online attacked sample detection process in Algorithm 2.
**Algorithm 2** Online attacked sample detection**Input** **:**Local poisoned data $D_{p}$, local benign data $D_{c}$, suspicious pre-trained DNN model *f*.1:Use $D_{p}$ and $D_{c}$ to fine-tune the *f*2:**for** all $x\in D_{p}$ **do**3:    $A_{x}$← Deep features of the poisoned data extracted by model *f*4:    Add $A_{x}$ to the backdoor feature set $A_{p}$5:**end for**6:**for** all $x\in D_{c}$ **do**7:    $A_{x}$← Deep features of the benign data extracted by model *f*8:    Add $A_{x}$ to the benign feature set $A_{c}$9:**end for**10:Train $f_{c}$ using $A_{p}$ and $A_{c}$11:$A_{x}$←Deep features of the input sample *x* extracted by the online deployment model *f*12:**if**$f_{c}\left(A_{x}\right)=benign$**then**13:    Permission *x* input *f*14:**else**15:    Reject *x* input *f*16:**end if**

## 4. Offline Defense Experiment Results and Analysis

### 4.1. Datasets and Models

For the sentiment analysis task, SST-2 was chosen as the dataset for the experiment [27]. It contains 6920 training samples, 872 validation samples, and 1821 test samples. For the hate speech detection task, HateSpeech (HS) is a typical hate speech dataset [28]. It is divided into two classes: clean and hate. One of the classes was randomly selected as the target class in the experiment. Two advanced, pre-trained language model models for processing NLP tasks were chosen as target models. They were BERT [5] and ALBERT (albert-base-v1) [6], and are both based on the transformer structure [29].

### 4.2. Attack Methods and Baseline Defense Methods

Char-level [9]. This method is a character-level backdoor attack method. The backdoor attack is launched by modifying the word in the sample to the specified trigger word by controlling the character editing distance.

BadNet-RW [10]. This method is a word-level backdoor attack method that launches a backdoor attack by adding a specified rare trigger word to the sample.

InsertSent [11]. This method is a sentence-level backdoor attack method that launches a backdoor attack by adding a specified trigger sentence to the sample.

AC [17] was selected as the baseline defense method based on scenario 1. This method is used to stop backdoor attacks by detecting poisoned samples in suspicious training data. Its required conditions and applicable scenarios are similar to those of the method in this chapter, so it is used as a baseline defense method. The specific techniques used in this method include the PCA dimensionality reduction technique and K-means clustering technique. In the experiments, PCA was first used to reduce the dimensionality to 10 dimensions, and then k-means clustering was used to divide the data into two classes. The class with less data was marked as a poisoned sample.

### 4.3. Experimental Settings

Regarding the scenario where the user trains the NLP model locally using the untrustworthy dataset directly, at this point, the defender has the complete suspect training dataset (which includes the complete benign training samples and the attacker-constructed poisoned training samples), the NLP model, and a small amount of known benign data. For the SST-2 dataset, both the attacker-constructed training sample size and defender-constructed poisoned training sample size are 10% of the original SST-2 dataset. The attacker-constructed poisoned samples are unknown and the defender-constructed poisoned samples are known. The NLP model is first trained with the known poisoned samples together with the suspicious dataset. Then, features are extracted using the NLP model. Regarding the training set and test set division methods of the classifier, the extracted known benign sample features and known poisoned sample features are used to train the classifier. The performance of the classifier is evaluated on the suspicious class benign training data and the unknown poisoned training data. For the HateSpeech dataset, both the attacker and the defender construct a poisoned training sample size of 2% of the original HateSpeech dataset. The NLP model is first trained with the known poisoned samples together with the suspicious dataset. Then, features are extracted using the NLP model. Regarding the training set and test set division methods of the classifier, the extracted known benign sample features and known poisoned sample features are used to train the classifier. The performance of the classifier is evaluated on the suspicious class benign training data and the unknown poisoned training data.

### 4.4. Defense Evaluation Metrics

The classification accuracy and $F_{1}$ value of the suspicious sample set are used as evaluation metrics. The classifier makes a judgment on whether the samples in the suspicious dataset are poisoned samples or not. If a sample is determined to be poisoned, a ‘Positive’ decision is made. *TP* (True Positive) indicates the number of ‘Positive’ determinations made by the classifier and the number of correct determinations. Similarly, the value of *FP* (False Positive) indicates the number of incorrect ‘Positive’ determinations. The value of *TN* (True Negative) indicates the number of correct ‘Negative’ determinations. The value of *FN* (False Negative) indicates the number of incorrect ‘Negative’ determinations. The accuracy (*ACC*) expression is as follows.

(10) $$\begin{array}{c} ACC=\frac{TP+TN}{TP+TN+FP+FN} \end{array}$$

*Precision* is the probability of being ‘Positive’ among all of the samples that are predicted to be ‘Positive’. The expression is as follows.

(11) $$\begin{array}{c} Precision=\frac{TP}{TP+FP} \end{array}$$

*Recall* is for the original sample and means the probability of being predicted as ‘Positive’ in a sample that is actually ‘Positive’. The expression is as follows.

(12) $$\begin{array}{c} Recall=\frac{TP}{TP+FN} \end{array}$$

The $F_{1}$ value is a combined measure of accuracy and recall and is expressed as follows.

(13) $$\begin{array}{c} F_{1}=2\cdot \frac{Precision\cdot Recall}{Precision+Recall} \end{array}$$

Because it is a binary classification experiment, we only calculated the $F_{1}$ values of ‘Positive’ samples, i.e., $F_{1}$ values of poisoned samples.

### 4.5. Defending Performance

The experiments evaluated the detection ability of two defense methods on suspicious training data. In the experiments, the defender chose “comparatively” as the trigger to poison small local benign datasets to generate known poisoned samples, and used these poisoned samples and suspicious training datasets to train BERT and ALBERT models. The poisoned samples constructed by the defender are not the same as those of the backdoor attacker. Table 2 shows the detection results of the defense method on the suspicious samples in the SST-2 training dataset. It can be seen that our method (DFC) has good defense against a variety of backdoor attacks when applied to two popular NLP pre-training models. In particular, the $F_{1}$ value of poisoned training samples detection reaches 100% in the face of InsertSent attack ALBERT. The experimental results prove the effectiveness of the method in this chapter. The detection performance of the baseline method (AC) fluctuates frequently. This is because the small proportion of poisoned samples in the suspicious training dataset increases the difficulty of clustering. On the other hand, the close distance between the two types of feature distribution also leads to the poor detection performance of the baseline method.

Table 3 shows the detection results of the defense methods on suspicious samples in the HateSpeech training dataset. It can be seen that our method has the same good backdoor defense effect on the hate speech detection task. It shows that the defense method is applicable to a wide range of NLP tasks and models.

## 5. Online Defense Experiment Results and Analysis

### 5.1. Attack Methods and Baseline Defense Methods

The online defense effectiveness of the proposed methods was evaluated on four backdoor attack methods. Three of the attack methods, Char-level, BadNet-RW, and InsertSent, only require poisoning a small amount of training data to achieve backdoor embedding and do not require the attacker to control the DNN model training process and modify the DNN model structure. The specific methods are consistent with the offline defense experiments and will not be described here. Embedding poisoning (EP) [15] is an attack method that modifies only the individual word embedding associated with a trigger during the backdoor injection process. It requires the attacker to control the training process of the model. It is a backdoor threat faced by users in scenarios where they use third-party training models.

Based on scenario 2, RAP was chosen as the baseline defense method in this section [30]. The backdoor defense method AC is not applicable to this scenario. RAP detects attacked samples online using the difference in robustness between the attacked and benign samples. A rare word is selected and only its word embedding parameters are manipulated to generate a perturbation. The perturbation is added to the benign sample and the model output probability of the target class decreases above a threshold. Adding the perturbation to the attacked sample, the model output probability of the target class decreases less than the threshold.

### 5.2. Experimental Settings

For the scenario where the user uses a third-party pre-trained NLP model, the defender has no control over the pre-training process and does not have access to the complete training data. At this point, the defender holds a small benign dataset to fine-tune the target model. For the SST-2 dataset, the attacking side constructs a poisoned training sample size of 10% of the original SST-2 dataset. The defender-constructed poisoned training sample size is 5% of the original SST-2 dataset. The attacker-constructed poisoned samples are unknown and the defender-constructed poisoned samples are known. Because the defender does not have the complete training data, the NLP model is first fine-tuned with a small benign dataset and known poisoned samples. Then, the features of the NLP model output are extracted. The extracted known benign sample features and known poisoned sample features are used to train the classifier. Regarding the training set and test set division methods of the classifier, the poisoned training samples features and a small number of benign samples features constructed by the defender are the training set for the classifier, and the 400 attack test samples features and 600 benign test samples features constructed by the attacker are the test set for the classifier. For the HateSpeech dataset, the attacker constructs a poisoned training sample size of 2% of the original HateSpeech dataset. The defender-constructed poisoned training sample size is 1% of the original HateSpeech dataset. The poisoned samples constructed by the attacker are unknown and the poisoned samples constructed by the defender are known. Because the defender does not have the complete training data, the NLP model is first fine-tuned with a small benign dataset and known poisoned samples. Then, the NLP model is used to extract features. The extracted features of known benign samples and features of known poisoned samples are used to train the classifier. Regarding the training set and test set division methods of the classifier, the poisoned training samples features and a small number of benign samples features constructed by the defender are the training set for the classifier, and the 400 attack test samples features and 600 benign test samples features constructed by the attacker are the test set for the classifier.

### 5.3. Defending Performance

The experiments evaluated the ability of the defense methods in this chapter to detect attacked samples online. Table 4 demonstrates the effectiveness of the defense method applied to the SST-2 sentiment analysis task. Table 5 shows the effectiveness of the defense method applied to the HateSpeech hate speech task. In the experiments, our method (DFC) selected “comparatively” as the trigger to poison small local benign datasets to generate known poisoned samples. These samples were used to fine-tune the infected BERT and infected ALBERT models. “mb” was selected as the perturbation word for the baseline approach (RAP). The word embedding parameters of “mb” in the infected BERT and infected ALBERT models were manipulated. The experimental results show that DFC has a good online detection accuracy and $F_{1}$ values for all four backdoor attacked samples. Specifically, the proposed defense method is more effective in defending against Char-level, BadNet-RW, and InsertSent than against EP. This is because Char-level, BadNet-RW, and InsertSent embed the backdoor into the model by changing all of the weight parameters of the model, whereas EP only modifies the single word embedding associated with the trigger to embed the backdoor into the model. As a result, the distinction between attack samples and benign samples generated by EP is reduced in the depth features in the posterior layer of the model. For EP, the proposed defense method still has a good detection capability, the detection accuracy of the proposed defense method is always above 88%, and the $F_{1}$ value is always above 85% on different datasets and models. The experimental results prove the effectiveness of the proposed method. The baseline method also shows a good online detection performance in most cases. However, the attacked samples are not robust against the attack when the attacker only changes the trigger word embedding by EP or adds a long trigger with BadNet-RW. This makes the output probability significantly lower after adding “mb” as well, resulting in an inability to accurately distinguish attacked samples from benign samples.

### 5.4. Detailed Attack Results

**Figure 3:**
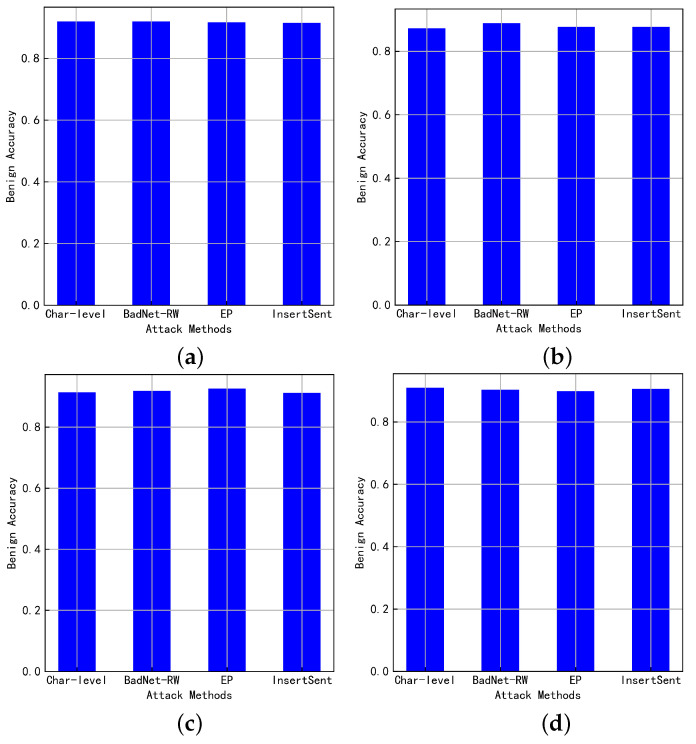
The benign accuracy of the backdoor model. (**a**) SST-2+BERT; (**b**) SST-2+ALBERT; (**c**) HS+BERT; (**d**) HS+ALBERT.

Figure 3 shows the benign accuracy of the NLP model after the backdoor is injected by the four attack methods. It can be seen that the NLP models injected with backdoors still have a very good benign accuracy. This makes the backdoor attack extremely stealthy. This is because it is difficult for users to perceive the difference in model performance without launching a backdoor attack.

**Figure 4:**
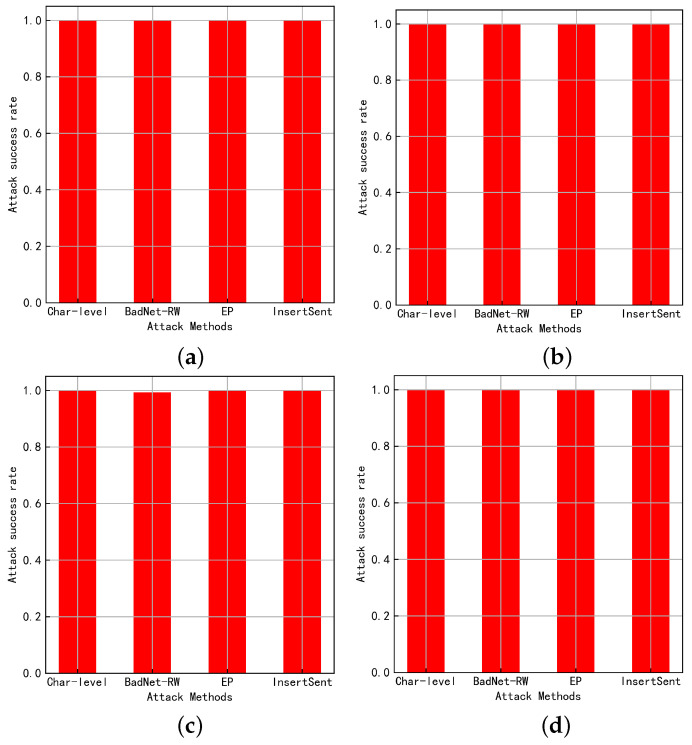
Attack success rate of the attack method. (**a**) SST-2+BERT; (**b**) SST-2+ALBERT; (**c**) HS+BERT; (**d**) HS+ALBERT.

Figure 4 shows the attack performance of the four attack methods on different tasks on the NLP model. It can be seen that, when the attacker launches a backdoor attack by adding triggers to benign samples, almost all achieve a 100% attack success rate. This indicates that the NLP model is extremely vulnerable to backdoor attacks, making the deployment of NLP models in risky application scenarios a great security risk.

## 6. Conclusions

In this work, we exploited the difference in deep features between poisoned and benign samples to propose a textual backdoor defense method DFC based on deep feature classification that is applicable to both offline and online defense scenarios. The experimental results show that DFC achieves excellent defense results in a variety of NLP tasks and models, and outperforms existing baseline defense methods.

## Figures and Tables

**Table 1:** The scenarios and corresponding attacker’s and defender’s capacities.

Scenarios	Attackers			Defenders			
	Training Data	Training Schedule	Model	Training Data	Training Schedule	Model	Inference Pipeline
Scenario 1	**✓**	** **	** **	**✓**	**✓**	**✓**	**✓**
Scenario 2	**✓**	**✓**	**✓**	** **	** **	**✓**	**✓**

**Table 2:** Performance of all offline defense methods in a sentiment analysis task.

Dataset	Attack	Defense	BERT		ALBERT	
			ACC	$F_{1}$	ACC	$F_{1}$
SST-2	Char-level	AC	53.30%	42.55%	99.83%	99.50%
		DFC	98.95%	97.05%	98.85%	96.73%
	BadNet-RW	AC	48.05%	29.17%	56.65%	44.37%
		DFC	99.88%	99.64%	99.68%	99.07%
	InsertSent	AC	53.37%	42.55%	99.45%	98.44%
		DFC	99.93%	99.78%	100%	100%

**Table 3:** Performance of all offline defense methods in a hate speech detection task.

Dataset	Attack	Defense	BERT		ALBERT	
			ACC	$F_{1}$	ACC	$F_{1}$
HS	Char-level	AC	92.85%	85.71%	99.21%	97.24%
		DFC	99.01%	96.58%	99.50%	98.26%
	BadNet-RW	AC	72.10%	50.09%	99.50%	98.26%
		DFC	99.70%	98.95%	99.50%	98.23%
	InsertSent	AC	84.11%	61.90%	22.84%	26.63%
		DFC	97.72%	92.10%	99.6%	98.60%

**Table 4:** Performance of all online defense methods in a sentiment analysis task.

Dataset	Attack	Defense	BERT		ALBERT	
			ACC	$F_{1}$	ACC	$F_{1}$
SST-2	Char-level	RAP	98.0%	98.40%	96.4%	97.08%
		DFC	100%	100%	99.1%	98.88%
	BadNet-RW	RAP	97.4%	97.92%	86.4%	87.98%
		DFC	93.1%	91.41%	95.8%	94.93%
	EP	RAP	97.7%	98.26%	86.8%	89.07%
		DFC	89.5%	86.02%	88.6%	85.31%
	InsertSent	RAP	72.4%	71.43%	89.6%	90.74%
		DFC	93.5%	91.20%	97.9%	97.42%

**Table 5:** Performance of all online defense methods in a hate speech detection task.

Dataset	Attack	Defense	BERT		ALBERT	
			ACC	$F_{1}$	ACC	$F_{1}$
HS	Char-level	RAP	97.9%	98.88%	98.4%	99.13%
		DFC	99.0%	98.76%	99.9%	99.88%
	BadNet-RW	RAP	98.9%	99.38%	98.4%	99.13%
		DFC	98.1%	97.63%	99.5%	99.38%
	EP	RAP	98.6%	99.25%	80.2%	88.39%
		DFC	97.7%	97.08%	94.3%	92.59%
	InsertSent	RAP	59.6%	71.81%	44.0%	56.59%
		DFC	96.0%	95.24%	99.5%	99.38%

## Data Availability

Data used in this research are from publicly available sources (https://nlp.stanford.edu/sentiment/, accessed on 28 November 2022, https://github.com/Vicomtech/hate-speech-dataset, accessed on 28 November 2022).
